# Unveiling the Influence of Copy Number Variations on Genetic Diversity and Adaptive Evolution in China’s Native Pig Breeds via Whole-Genome Resequencing

**DOI:** 10.3390/ijms25115843

**Published:** 2024-05-27

**Authors:** Haonan Yuan, Wenjing Wei, Yue Zhang, Changwen Li, Shengguo Zhao, Zhe Chao, Changyou Xia, Jinqiang Quan, Caixia Gao

**Affiliations:** 1 College of Animal Science and Technology, Gansu Agricultural University, Lanzhou 730030, China; hnyuan1101@163.com (H.Y.); weiwenjing@163.com (W.W.); 15377151933@163.com (Y.Z.); zhaosg@gsau.edu.cn (S.Z.); 2 State Key Laboratory for Animal Disease Control and Prevention, Harbin Veterinary Research Institute, Chinese Academy of Agricultural Sciences (CAAS), Heilongjiang Provincial Key Laboratory of Laboratory Animal and Comparative Medicine, National Poultry Laboratory Animal Resource Center, Harbin 150069, China; lichangwen@caas.cn (C.L.); xiachangyou@caas.cn (C.X.); 3 Institute of Animal Science and Veterinary Medicine, Hainan Academy of Agricultural Sciences, Key Laboratory of Tropical Animal Breeding and Disease Research, Haikou 571100, China; chaozhe@hnaas.org.cn

**Keywords:** genomic analysis, selective breeding, gene–environment interaction, swine genomics, animal model applications

## Abstract

Copy number variations (CNVs) critically influence individual genetic diversity and phenotypic traits. In this study, we employed whole-genome resequencing technology to conduct an in-depth analysis of 50 pigs from five local swine populations [Rongchang pig (RC), Wuzhishan pig (WZS), Tibetan pig (T), Yorkshire (YL) and Landrace (LR)], aiming to assess their genetic potential and explore their prospects in the field of animal model applications. We identified a total of 96,466 CNVs, which were subsequently integrated into 7112 non-redundant CNVRs, encompassing 1.3% of the swine genome. Functional enrichment analysis of the genes within these CNVRs revealed significant associations with sensory perception, energy metabolism, and neural-related pathways. Further selective scan analyses of the local pig breeds RC, T, WZS, along with YL and LR, uncovered that for the RC variety, the genes *PLA2G10* and *ABCA8* were found to be closely related to fat metabolism and cardiovascular health. In the T breed, the genes *NCF2* and *CSGALNACT1* were associated with immune response and connective tissue characteristics. As for the WZS breed, the genes *PLIN4* and *CPB2* were primarily linked to fat storage and anti-inflammatory responses. In summary, this research underscores the pivotal role of CNVs in fostering the diversity and adaptive evolution of pig breeds while also offering valuable insights for further exploration of the advantageous genetic traits inherent to China’s local pig breeds. This facilitates the creation of experimental animal models tailored to the specific characteristics of these breeds, contributing to the advancement of livestock and biomedical research.

## 1. Introduction

Copy number variations (CNVs) constitute significant structural genetic alterations that encompass key regions of the genome, varying in size from 50 kb to several Mb. These variants, which include duplications, insertions, and deletions, fill the genetic gap between single nucleotide polymorphisms (SNPs) and large-scale chromosomal rearrangements [1,2]. As critical biomarkers, CNVs are implicated in a wide array of phenotypic diversity, disease susceptibility, adaptability, and production traits among various domesticated species, such as cattle (*Bos taurus*) [3], sheep (*Ovis aries*) [4], pigs (*Sus scrofa*) [5], chickens (*Gallus gallus*) [6], and dogs (*Canis familiaris*) [7]. For instance, research conducted by Bovo et al. on 19 European local pig breeds and two commercial breeds (Italian Large White and Italian Duroc) revealed that a CNVR within the KIT gene correlates with the coat color phenotype of the pigs examined, while a CNVR encompassing the *MSRB3* gene is related to ear size [8]. Repetitions in the coding region of the Agouti Signaling Protein (*ASIP*) gene in sheep may be linked to variations in pigmentation [9]. During the domestication of dogs, an increase in the copy number of the *AMY2B* gene has bolstered their ability to digest foods rich in starch [10]. In Leizhou black goats, variations in the copy number of the plectin (*PLEC*) gene are intimately connected with growth and muscle development, indicating the potential of this gene as an auxiliary marker for goat breeding selection [11]. Additionally, advances in genomic technologies have catapulted whole-genome resequencing (WGRS) to prominence, rendering SNP chips and array comparative genomic hybridization (CGH) less effective by comparison [12,13]. This delivers a higher resolution of CNV detection and genotyping, deepening our understanding of the functional genetics behind animal breeding strategies and the mechanisms of disease pathogenesis.

With the integration of modern biotechnology and the livestock industry, commercial pig breeds like Yorkshire (YL) and Landrace (LR), known for their efficient growth and feed conversion ratios, have been widely adopted, significantly boosting the economic benefits of pig farming. However, this homogenized breeding strategy has overshadowed local pig breeds, such as the Rongchang pig (RC), Tibetan pig (T), and Wuzhishan pig (WZS). Despite slower growth and lower yields, these local breeds demonstrate unique advantages in disease resistance, reproductive efficiency, and adaptability to harsh environments, and their meat also boasts distinctive flavors [14,15,16]. Additionally, local pig breeds hold considerable advantages as experimental models due to their genetic diversity and adaptability, which are vital for researching disease models relevant to humans [17]. For example, the potential of RC pigs in metabolic and obesity research, as well as the value of T pigs in immune mechanism studies, are noteworthy [18,19]. These characteristics position local pigs as key players in understanding genetic diseases, developing new treatments, and expanding the repertoire of experimental models to enhance the applicability of health research to humans. Concurrently, such research fosters the conservation and sustainable use of genetic resources, helping to overcome the limitations of existing models and laying a new foundation for translational clinical research.

Therefore, to accurately assess the genetic potential of these local pig breeds and explore their prospects in the field of animal models, this study has selected SPF (Specific Pathogen Free) YL and LR pigs to ensure a highly controlled research environment and the accuracy of experimental data. We will employ WGRS technology to meticulously analyze the differences in CNVs between these local pig breeds and commercial strains and to reveal genetic variations associated with their unique traits. Furthermore, these findings will contribute to establishing the status of local pig breeds as animal models in experimental medical research, providing valuable genetic background information for studies on disease mechanisms and the development of new drugs.

## 2. Results

### 2.1. Genome-Wide Assay Results for CNVs

**Figure 1:**
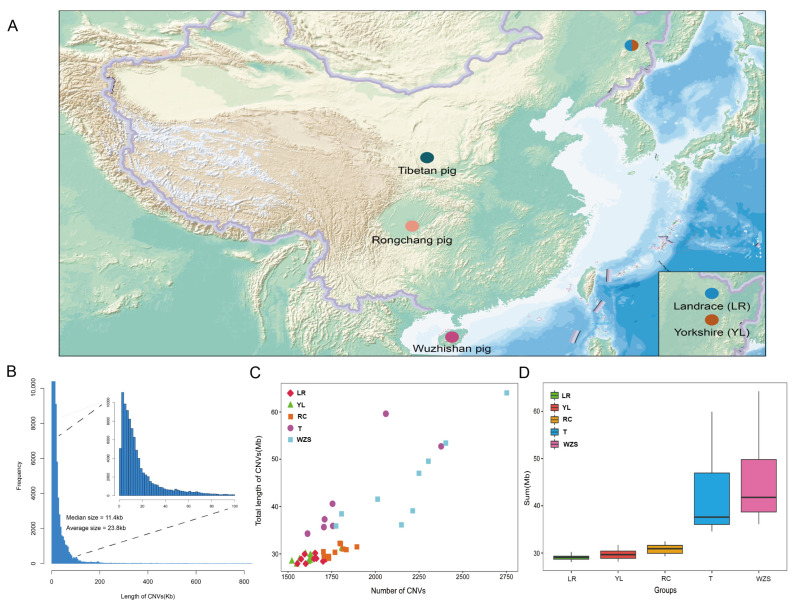
Geographical distribution of local and commercial pig breeds and whole-genome characterization of CNVs in the porcine genome. (**A**) A map illustrating the geographic distribution of the five pig breeds sampled in this study. Dashed circles represent the Harbin Veterinary Research Institute, the breeding ground for imported commercial pig breeds, as shown in the lower right corner, namely YL and LR pigs. (**B**) A histogram of CNV length distribution. (**C**) The total length and number of CNVs identified in each sample. (**D**) Boxplots depicting the distribution of CNV lengths within each group.

Using the Illumina NovaSeq 6000 sequencing platform, we constructed short-read sequencing libraries for 50 pigs, obtaining a total of 26.62 Tb of DNA base pairs, with an average sequencing depth of 9.51× per individual, ensuring the reliability of CNV detection (Supplementary Table S2). Employing the CNVcaller software (V. 1.1), we identified a total of 96,466 CNVs across all autosomes, which included 52,418 duplication events and 42,248 deletion events. The size distribution of CNVs exhibited an “L” shaped curve, with a median size of 11.4 kb and an average size of 23.4 kb (Figure 1B). Additionally, violin plots demonstrated that the distribution of CNV lengths varied among different pig breeds, with the local breeds RC, T, and WZS having a higher total number of CNVs compared to the introduced commercial breeds YL and LR, with T and WZS showing particularly high variability within this group (Figure 1D). On an individual level, the porcine genome contained an average of 1929.32 CNVs, ranging from 1522 to 5709 (Figure 1C; Supplementary Table S3).

**Figure 2:**
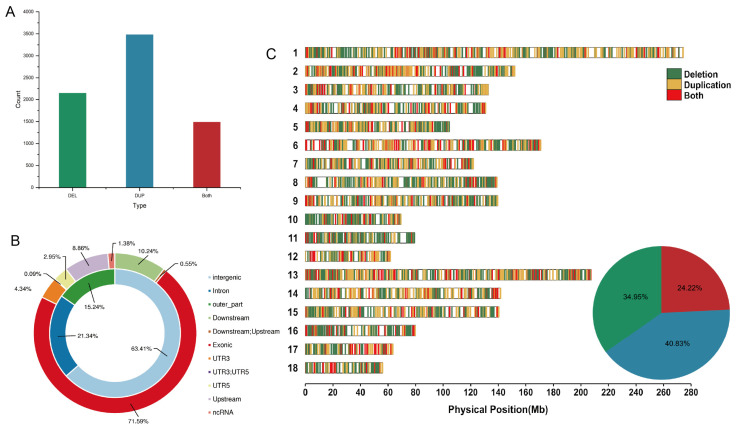
Genomic diversity and distribution of CNVRs. (**A**) The number of CNVRs detected. (**B**) Annotation of CNVRs with various genomic features. (**C**) A distribution map of CNVRs across the 18 autosomes and pie charts depicting the proportions of CNVRs with three different copy number states.

By integrating overlapping CNVs from five populations, we identified a total of 7112 CNVRs, comprising 2145 deletions, 3481 duplications, and 1486 both deletions and duplications, which account for 1.3% of the porcine reference genome (Figure 2A; Supplementary Table S4). In the distribution of CNVRs across chromosomes, the highest numbers of CNVRs were detected on chromosomes 1, 2, and 13, with totals of 684, 605, and 547, respectively (Figure 2C; Supplementary Table S5). Correlation analysis demonstrated a positive relationship between the number of CNVRs and the corresponding chromosome lengths (R^2^ = 0.81, *p* < 0.05) (Supplementary Figure S1). The annotation of CNVRs indicated that 4510 CNVRs were located in intergenic regions, 1518 in introns, and 776 in exons (Figure 2B).

### 2.2. Comparison with Other Studies of Known CNVs in Pigs

Our research was juxtaposed with four preceding studies focused on pig CNVRs referencing the Sscrofa11.1 assembly. These earlier works unveiled between 115 and 11,173 CNVRs, their extent stretching from 12.54 to 197.04 Mb. Notably, the CNVRs discerned in our investigation partially coincided with those documented earlier, evidencing overlap rates that fluctuated from 2% to 49%, and the overlapping segments ranged in length from 2.22 to 19.99 Mb (Table 1).

### 2.3. Annotation of CNVRs

Of the 7112 CNVRs identified, 2141 (35.91%) encompass 2548 protein-coding genes. These genes, which have undergone copy number variations, provide a highly valuable resource for investigating the relationship between CNV genes and phenotypic expression. To further study the functions of these CNVRs, we performed a functional enrichment analysis on the genes contained within the CNVRs. A total of 126 GO terms were significantly enriched (*p* < 0.01) across five pig populations, primarily including 15 cellular components, 44 molecular functions, and 67 biological processes. These GO terms were related to sensory perception systems (GO:0050911, GO:0007608, GO:0007600), receptor signaling activity (GO:0038023, GO:0004872), and energy metabolism (GO:0004617, GO:0033906) among others (Supplementary Table S6). KEGG pathway analysis revealed significant enrichment of CNVR-associated genes in 18 pathways (*p* < 0.05, Supplementary Table S7), including olfactory transduction (ko04740), metabolic pathways (ko00140, ko01040, ko00053), and processes associated with the nervous system (ko05033).

To further elucidate the correlations between CNVRs and swine traits, we downloaded the pig QTL data from the pig quantitative trait loci database (https://www.animalgenome.org/cgi-bin/QTLYL/SS/index (accessed on 20 March 2024)) and conducted a comparison with population CNVRs. Our findings reveal that 2239 CNVRs show overlap with 302 distinct pig QTL trait regions (Supplementary Table S8). These QTLs are predominantly associated with qualitative meat characteristics, including meat quality, color, tenderness, and the quantity characteristics of backfat. Notably, the QTLs overlapping with backfat include intramuscular fat content QTLs, saturated fatty acid QTLs, unsaturated fatty acid QTLs, and backfat thickness at the loin QTLs. This information is of significant value for the future genetic improvement of local pig breeds.

### 2.4. CNVRs Population Genetics

We applied the Vst statistic to analyze CNVRs among three local pig breeds and two imported commercial breeds, conducting pairwise Vst comparisons for RC with YL and LR, T with YL and LR, and WZS with YL and LR. This approach allowed us to assess the extent of CNVR variability among different groups. By employing an empirical distribution, we selected the top 5% of CNVRs based on their Vst values as candidate CNVRs. The results indicated that within the top 5% of Vst values, there were 135 CNVRs in the RC with YL and LR comparison, 136 CNVRs in T with YL and LR, and 128 CNVRs in WZS with YL and LR.

**Figure 3:**
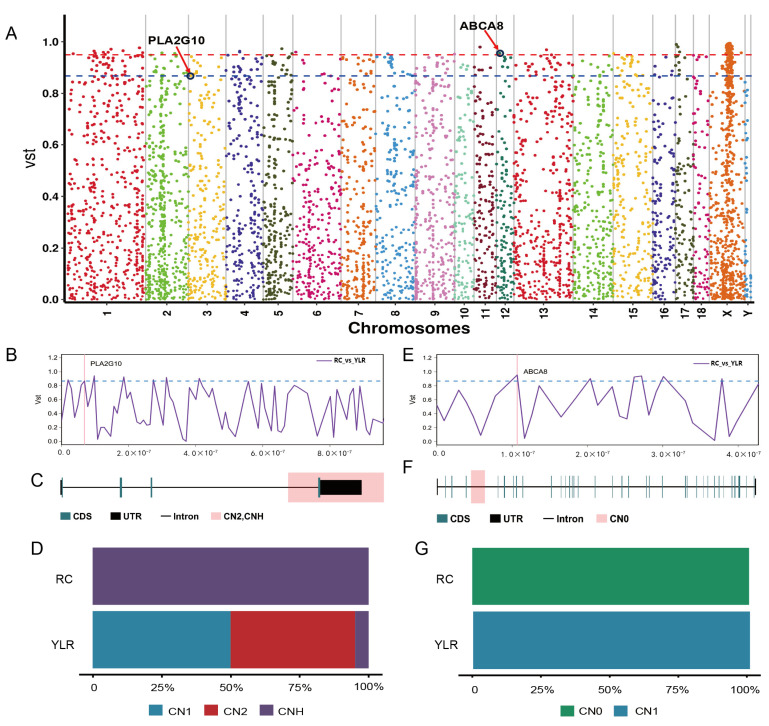
Comparative genomic analysis of RC with YL and LR pigs using Vst. (**A**) Manhattan plot of genome-wide Vst for each CNVR locus between RC, YL, and LR pigs. (**B**) An enlarged line plot of the PLA2G10 region. (**C**) Gene structure of PLA2G10. (**D**) Allele frequency distribution for PLA2G10. (**E**) An enlarged line plot of the ABCA8 region. (**F**) Gene structure of ABCA8. (**G**) Allele frequency distribution for ABCA8.

Among the 135 significantly differential CNVRs were identified between RC with YL and LR (Figure 3A). Functional enrichment analysis revealed that the genes overlapping with differentiated CNVRs were primarily involved in pathways such as Fat digestion and absorption (ko04975) and ABC transporters (ko02010) (Supplementary Table S10). The most notable loci were located on chromosomes 3 and 12, within the *PLA2G10* and *ABCA8* genes, respectively. These stratified loci include two CNVRs (3:7638801–7679600 and 12:11382001–11385200) characterized as duplication and deletion types (Figure 3B,C,E,F). The duplication frequency of the *PLA2G10* gene was completely fixed in RC pigs, while it only occurred at a frequency of 50% in YL and LR (Figure 3D). Deletion frequency within the *ABCA8* gene was only found in RC, with no variations observed in YL and LR (Figure 3G).

**Figure 4:**
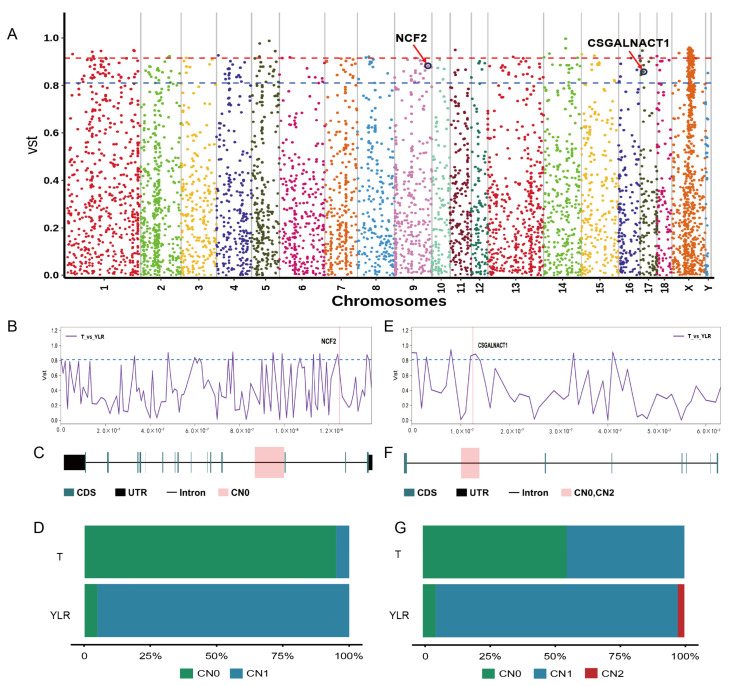
Comparative genomic analysis of T with YL and LR pigs using Vst. (**A**) Manhattan plot of genome-wide Vst for each CNVR locus between T, YL, and LR pigs. (**B**) An enlarged line plot of the NCF2 region. (**C**) Gene structure of NCF2. (**D**) Allele frequency distribution for NCF2. (**E**) An enlarged line plot of the CSGALNACT1 region. (**F**) Gene structure of CSGALNACT1. (**G**) Allele frequency distribution for CSGALNACT1.

Between T with YL and LR, significant differences were observed in the genes overlapping with the 136 CNVRs, with a notable enrichment in pathways such as Glycosaminoglycan biosynthesis—chondroitin sulfate/dermatan sulfate (ko00532) and Phagosome (ko04145) (Supplementary Table S11). Among all analyzed CNVRs, the NCF2 (Neutrophil Cytosolic Factor 2) gene (9:124795201–124798400) was found to be absent in T pigs at a frequency of 95%, while its absence in YL and LR was observed at a mere frequency of 5% (Figure 4A–D). In the case of the CSGALNACT1 (Chondroitin Sulfate N-Acetylgalactosaminyltransferase 1) gene, deletions were observed in the T group at a frequency of 55%, while in YL and LR, deletions and duplications coexisted but were less prevalent, with the majority showing no variation (Figure 4E–G). 

**Figure 5:**
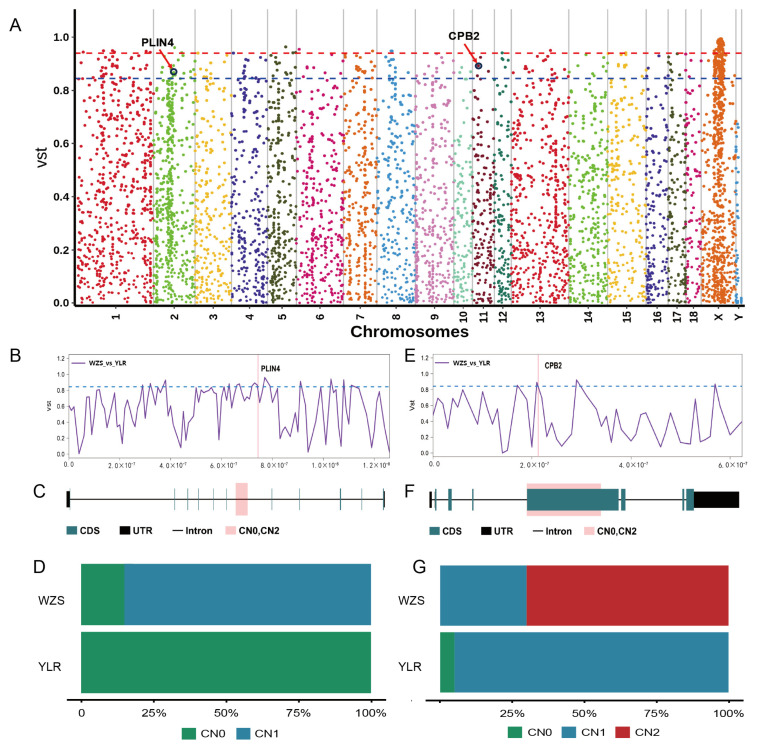
Comparative genomic analysis of WZS with YL and LR pigs using Vst. (**A**) Manhattan plot of genome-wide Vst for each CNVR locus between WZS, YL, and LR pigs. (**B**) An enlarged line plot of the PLIN4 region. (**C**) Gene structure of PLIN4. (**D**) Allele frequency distribution for PLIN4. (**E**) An enlarged line plot of the CPB2 region. (**F**) Gene structure of CPB2. (**G**) Allele frequency distribution for CPB2.

Between WZS with YL and LR, we identified significant population differentiation in genes overlapping with 128 CNVRs, predominantly involved in the PPAR signaling pathway (ko03320) (Supplementary Table S12). The most significant loci were found on chromosome 2 (2:74322801–74326000) and chromosome 11 (11:21268401–21270800), within the PLIN4 (perilipin 4) and CPB2 (carboxypeptidase B2) genes, respectively (Figure 5A). In WZS, *PLIN4* was identified as being of the deletion type but with a reduced deletion frequency of only 15%, while it was completely absent in YL and LR (Figure 5B–D). For the *CPB2* gene, the majority of WZS instances exhibited duplications of more than two copies, with virtually no variations in the YL and LR groups (Figure 5E–G), potentially playing a significant role in the adaptation of WZS pigs to their local environment.

### 2.5. qPCR Validation of CNVRs

To verify the accuracy of CNV predictions made by CNVcaller, we randomly selected 9 CNVRs for qPCR validation. The results indicated that the predictions for all selected CNVRs were confirmed by the CNVcaller’s analysis (Supplementary Figure S2).

## 3. Discussion

CNVs serve as a significant component of genomic sequence differences among individuals, potentially influencing phenotypic changes through alterations in gene structure, gene dosage, and the regulation of allele frequencies [22,23]. In this study, NGS technology was employed to detect CNVs in 50 pigs derived from five different populations. By merging overlapping CNVs detected within each sample, a total of 96,466 CNVs were identified. The number of CNV events per individual ranged from 1522 to 5709. Subsequently, aggregating these individual data points to obtain a non-redundant CNVR dataset resulted in 7112 CNVRs, comprised of 2145 copy number deletions and 3481 copy number duplications. Interestingly, our findings revealed that copy number duplication events were more common than deletion events, diverging from previous reports [24]. This discrepancy could be attributed to the specific genetic backgrounds or the unique populations of indigenous Chinese pigs studied, which may exhibit a higher propensity for copy number duplications in certain genomic regions due to natural selection or genetic drift.

Considering that the detection rate of CNVRs is influenced by various factors, we compared our research findings with previously reported CNVRs. This comparison revealed differences among the CNVRs discovered in these studies, which may be attributed to several causes. These include variations in pig breeds that affect the distribution and frequency of copy number variations, differing technologies and methods used (such as array hybridization, whole-genome sequencing, targeted region sequencing), CNV detection algorithms (e.g., PennCNV, CNVnator, BreakDancer), the number of samples included and the selection criteria of these samples (such as age, sex, health status), and the quality of genomic DNA extraction. These discrepancies underscore the complex nature of CNVR detection [25,26]. CNVs have profoundly influenced gene expression regulation, gene functionality, and phenotypic traits in organisms, representing a crucial source of genetic diversity [27,28]. GO enrichment analysis reveals that common CNVRs detected across five pig breeds are significantly enriched in GO terms related to sensory perception and energy metabolism. The enhancement of sensory perception aids these breeds in better detecting environmental food sources, mates, and potential threats, thereby increasing their reproductive success, is also significantly enriched in horses [29], sheep [30], and yaks [31]. Notably, olfaction plays a critical role in rapidly responding to environmental changes, with a strong representation in the gene families involved in sensory perception. The research by Paudel and others investigating the mechanisms of gene flow between evolving populations supports our findings, showing that the copy number variations formed in pig species might be related to olfactory receptors [32]. Regulation of energy metabolism is vital for pigs to thrive and reproduce under various climatic conditions and food resource availability. This likely reflects the evolutionary metabolic strategies that different pig breeds have developed to adapt to diverse living environments. For instance, Tibetan pigs inhabiting high-altitude areas might have evolved more efficient mechanisms for energy utilization to cope with hypoxic and cold conditions [33]. Interestingly, in our KEGG pathway analysis, the results are consistent with the GO enrichment findings, highlighting that the shared CNVRs are also significantly enriched in pathways related to the neural system. The neural system, as one of the most direct and rapid responders to external environmental changes, is essential for animal survival and adaptation [34]. Zhang et al. discovered that, compared to Chinese domestic pigs and Large White pigs, the identified Neural Glycoprotein 4 X-linked (NLGN4X) and Neural Glycoprotein 4 Y-linked (NLGN4Y) in Su-Huai pigs are associated with neurological diseases and neural signal transduction. This suggests that Su-Huai pigs are more docile and easier to domesticate than Large White pigs [21]. In enriched environments, swine have a higher level of proteins related to protein synthesis and neural activity, enabling them to better cope with stressful conditions like those found in slaughterhouses [35]. The enrichment of CNVR-overlapping genes in neural system-related signaling pathways provides a genetic basis for these pig breeds to adapt to varying environmental challenges. It supports their ability to cope through optimized neural responses and behavior. This enrichment not only deepens our understanding of the evolutionary mechanisms underpinning pig adaptability but also offers valuable insights for future research in genetic improvement and environmental adaptability of pig breeds.

Selective sweep analyses can reveal key candidate gene regions influenced by environmental and artificial selection during the adaptation and domestication processes [36]. In our study, we identified 135 significantly differentiated CNVRs through comparisons among RC, YL, and LR; functional enrichment analysis indicated that genes overlapping with these differentiated CNVRs are primarily involved in crucial biological pathways like fat digestion and absorption, and ABC transporters, suggesting their pivotal roles in the adaptive differences among these pig breeds. Notably, the *ABCA8* and *PLA2G10* genes, located in significantly differentiated CNVRs on chromosomes 3 and 12, respectively, exhibit deletion and duplication variations. Intriguingly, the duplication frequency of the *PLA2G10* gene is completely fixed in RC pigs, while it is only 50% in YL and LR, indicating a selection pressure in the RC population that favors this gene duplication. The *PLA2G10* gene, implicated in the selective hydrolysis of phospholipids, plays a vital role in fat metabolism and energy utilization. Such genetic adaptation may enhance the ability of RC pigs to cope with fluctuating food availability, thereby aiding their survival and thriving in variable environmental conditions [37,38,39]. Similarly, the deletion variation of the *ABCA8* gene is exclusively observed in RC pigs and absent in YL and LR, suggesting a unique role for this gene deletion in RC adapting to specific environmental conditions. The *ABCA8* gene is related to lipid metabolism and cardiovascular health; its absence may alter physiological mechanisms in these aspects for RC, providing them an adaptive advantage [40,41].

T, endemic to the high-altitude regions of China, possess unique adaptations to hypoxic conditions [42]. In our genetic comparison with YL and LR, we identified key molecular markers that may explain the survival and adaptive advantages of Tibetan pigs in specific environments. Notably, these marker genes are significantly enriched in pathways, including glycosaminoglycan biosynthesis and phagocytosis, highlighting the potential importance of these pathways in inter-breed adaptive differences. The *NCF2* and *CSGALNACT1* genes exhibit unique variation patterns in Tibetan pigs, which could have profound effects on their physiological and immune functions. Firstly, the *NCF2* gene, a protein found in neutrophils crucial for activating the NADPH oxidase [43,44,45,46], shows a 95% deletion rate in Tibetan pigs. This variation might alter the ability of neutrophils to produce reactive oxygen species via the NADPH oxidase, a key mechanism for the immune system to clear invading pathogens. This specific deletion variant could provide T pigs with an advantage in combating certain types of infections, especially those requiring strong reactive oxygen species-mediated immune responses. Moreover, the *CSGALNACT1* gene, responsible for the synthesis of chondroitin sulfate, displays a 55% deletion frequency in the Tibetan population, a figure that is particularly notable compared to YL and LR. Given the role of chondroitin sulfate in numerous biological processes, including cell signaling and intercellular interaction, this variation might significantly impact the connective tissue characteristics and immune response capabilities of Tibetan pigs [47,48]. In summary, these findings suggest that Tibetan pigs may have gained adaptive advantages in their high-altitude, low-oxygen environments through specific genetic variations.

In comparative analysis between WZS with YL and LR, we observed significant population differentiation associated with genes overlapping 128 CNVRs, particularly involving the PPAR signaling pathway (ko03320). This pathway plays a crucial role in lipid metabolism, energy balance, and inflammatory responses, underscoring its potential importance in breed-specific adaptations. Notably, we identified two key loci on chromosomes 2 and 11, which respectively harbor the *PLIN4* and *CPB2* genes. The *PLIN4* gene plays a significant role in regulating lipid metabolism and fat storage, especially in managing the formation and breakdown of lipid droplets in adipocytes [49]. In WZS, there is only a 15% deletion rate of the *PLIN4* gene, in contrast to a 100% deletion rate in both YL and LR. Living in environments where resources are scarce, the ability to effectively store and utilize fat is key to survival and reproductive success. Compared to YL and LR, which completely lack the *PLIN4* gene, WZS may better maintain energy balance under conditions of unstable food supply, reflecting a genetic advantage in adapting to their specific habitats. On the other hand, the *CPB2* gene encodes carboxypeptidase B2, which plays a critical role in blood coagulation and inflammatory responses [50]. In WZS, there is a high rate of *CPB2* gene copy number duplication (70%), as opposed to a much lower occurrence in YL and LR (only 5% deletion). In the wild, animals frequently encounter physical injuries or pathogenic attacks. A higher expression level of the *CPB2* gene could accelerate the blood coagulation process, reduce bleeding time from wounds, and may also facilitate rapid initiation and effective control of inflammatory responses, which are crucial for preventing infections and accelerating wound healing [51]. Moreover, an increase in *CPB2* gene copy number could enhance resistance to certain diseases. In inflammatory responses, the activity of carboxypeptidase B2 is important for regulating immune responses, promoting the repair of damaged tissue, and limiting the spread of pathogens [52]. Therefore, higher expression of *CPB2* might provide WZS with stronger physiological defense mechanisms when facing pathogens in their environment. This genetic characteristic reflects the evolutionary adaptation of WZS to their specific living conditions, holding significant value for the conservation and utilization of this local breed.

## 4. Materials and Methods

### 4.1. Ethics Statement

All animal work was conducted following the guidelines for the care and use of laboratory animals established by the Chinese Ministry of Agriculture. In addition, the research was carried out in accordance with the regulations set by the Ethics Committee of the Harbin Veterinary Research Institute, Chinese Academy of Agricultural Sciences (NO: 211008-02), ensuring compliance with ARRIVE’s guidelines and standards.

### 4.2. Whole-Genome Sequencing Data Analysis

In this study, fresh blood samples were collected from 50 pigs, which included three native breeds ((RC (5♀, 5♂)), (T (5♀, 5♂)), and (WZS (5♀, 5♂))), and two SPF-grade commercial breeds introduced from Canada (YL (5♀, 5♂)) and (LR (5♀, 5♂)). The samples for the RC, T, and WZS pig populations were sourced from Chongqing, Gannan, and Hainan in China, respectively. The imported SPF-grade YL and LR were reared in a sterile environment specifically provided by the National Experimental Animal Resource Management Center in Harbin, China (Figure 1A; Supplementary Table S1). All pigs were adult pigs. Following the extraction of genomic DNA from the samples, genomic DNA was randomly sheared into approximately 350 bp fragments using ultrasound according to the TruSeq Nano DNA HT Library Prep Kit instructions. The fragmented DNA is end-repaired to form blunt ends, then A-tailed, and additional adenine bases are added to facilitate attachment of DNA fragment adapters. After adapter ligation, the DNA is PCR amplified and purified to produce the final DNA library. Sequencing was performed using the NovaSeq 6000 (Illumina Inc., San Diego, CA, USA) platform at Genedenovo Biotechnology Co. (Guangzhou, China). The raw reads were processed according to stringent filtering criteria: (1) removal of reads with ≥10% unidentified nucleotides (Ns), (2) removal of reads where >50% of bases had a Phred quality score ≤20, (3) removal of reads aligned with adapter sequences. Subsequently, alignments to the pig reference genome (Sus scrofa 11.1) were carried out using the BWA (Burrows–Wheeler Aligner) software (V. 0.7.12). Binary alignment files generated by BWA were then sorted and indexed by scaffold positional information using SAMTOOLS software (V. 1.17) [53]. To enhance the accuracy of CNV detection, duplicate reads were removed from the original alignment using Picard software (http://broadinstitute.github.io/picard/ (accessed on 20 March 2024), V. 2.10.6). Furthermore, the Realigner Target Creator module within GATK software (V. 3.8.0) [54] was used for realigning sequences near InDels.

### 4.3. CNV Detection and CNVR Definition

We performed CNV detection and determined CNVRs using the CNVcaller software (V. 1.1) [55]. The reference genome Sus scrofa 11.1 was utilized and segmented into 800 bp windows to construct the reference genome database. Subsequently, the read counts per window in the BAM files, as well as the mapping reads, were computed. Reads with high similarity (≥97%) were merged, and regions of low complexity were removed. GC-content-based correction and normalization were conducted using the accurate read counts in each merged window to calculate absolute copy numbers. Preliminary boundaries of CNVRs were determined based on the distribution of absolute copy numbers, the frequency of variation, and the significant correlation between adjacent windows. Subsequently, copy number distributions and neighboring CNVRs that were significantly correlated with the population were further merged, yielding the final CNVR detection results. The resulting set of merged CNVRs was analyzed to calculate the allele frequencies of deletions and duplications for each individual. The allele frequencies of different CNVR types were screened under specific conditions: (1) For deletions (Loss): 0.05 < Loss AF ≤ 0.95 and Gain AF ≤ 0.01; (2) for duplications (Gain): 0.05 < Gain AF < 0.95 and Loss AF ≤ 0.01; (3) for complex types (Both): 0.05 < Gain AF < 0.95 and 0.05 < Loss AF ≤ 0.95. In addition, we refined the CNVR dataset by applying filters based on silhouette score and CNVR length: (1) Silhouette score > 0.6; (2) length of deletion-type CNVRs not exceeding 50 kb, duplication-type CNVRs not exceeding 500 kb, and complex-type CNVRs not exceeding 50 kb.

### 4.4. Comparison with Recent Reports

To ensure the precision of our research outcomes, we compared our results with those from four published studies on porcine CNVs that also referenced the Sus scrofa 11.1 genome. Subsequently, we identified the overlapping loci using the positional information of the CNVR intervals and then quantified the total number of CNVRs discovered, as well as the aggregate length of these variants.

### 4.5. Annotation of CNVR

After aligning all CNVRs to the physical positions on the swine reference genome, we retrieved CNVR-associated genes from the Ensembl database using the BioMart software (http://www.biomart.org (accessed on 20 March 2024)), retaining genes that were either fully or partially (≥50%) overlapping for subsequent analyses. Gene enrichment analysis for KEGG pathways and GO terms was performed using the online tool KOBAS 3.0 (http://kobas.LRi.pku.edu.cn/ (accessed on 20 March 2024)) [56], with a significance enrichment threshold set at *p*-value < 0.05. Moreover, to investigate whether these CNVRs are associated with important economic traits in pigs, we compared the CNVRs with QTLs listed in the Pig QTL database (https://www.animalgenome.org/QTLYL/pig.html (accessed on 20 March 2024)).

### 4.6. Population Genetics of CNVRs

To more accurately detect CNVRs that exhibit significant differentiation between populations, we employed an analysis similar to Fst known as Vst [57], which measures the variation in CNVRs among different breeds. The Vst is calculated using the formula: Vst = (Vt − Vs)/Vt, where Vt represents the standard deviation of the copy number size for a specific region across all samples, and vs. is the weighted average of the standard deviations calculated for the two sample groups based on their respective sizes. By computing the pairwise Vst values for CNVRs between the RC, T, WZS, and YL, LR groups, we focused on the top 5% of CNVRs with exceptionally high Vst values and explored whether these “outlier” loci are associated with significant swine traits. We subsequently defined the top 5% of CNVRs as regions of marked differentiation between the two groups and conducted a functional enrichment analysis on candidate genes within these overlapping regions.

### 4.7. qPCR Validation of CNVRs

To verify the accuracy of CNVR predictions, we randomly selected 9 CNVRs predicted by the CNVcaller software, including both Gain and Loss types, for qPCR validation. We downloaded the porcine reference gene sequences from the NLRI database based on the mutation loci predicted by the software, designed primers using Primer 5.0 software, and selected the *GCG* gene [58] as the internal reference gene (Supplementary Table S9). We carried out the detection on a LightCycler 480 II real-time PCR system, running each sample in triplicate to ensure the precision of quantification, and employed the 2^−ΔΔCt^ method to calculate the copy numbers of the target genes.

## 5. Conclusions

Our study of CNVs in 50 pigs has identified significant genetic variations associated with sensory, metabolic, and neural pathways. Specific genes across different breeds were found to influence essential functions such as metabolism, cardiovascular health, immune responses, and tissue characteristics. These insights improve our understanding of pig genomics, connecting CNVs to pivotal traits related to metabolism, immunity, and neurology. The data not only inform breeding strategies but also provide valuable perspectives for both agricultural methodologies and biomedical research.

## Figures and Tables

**Table 1:** Comparison of our study with four recent reports of porcine CNV using various platforms.

Study	Platform	Breed	Sample	CNVRs Number	Total Length (Mb)	Overlap Number	Overlap Ratio	Overlap Length (Mb)
Ding et al. (2022) [20]	SNP50 Bead	Duroc pigs	3770	695	174.43	446	0.06	2.35
Zhang et al. (2022) [21]	Illumina Hiseq2000	Suhuai pigs; Min Zhu; Large White pigs	46	11,173	44.97	3506	0.49	19.99
Panda et al. (2023) [5]	60 K SNP BeadChip	Landlly pigs	69	115	12.54	155	0.02	3.01
Stafuzza et al. (2019) [8]	SNP80 BeadChip	Lithuanian Indigenous Wattle; Turopolje pig	3892	425	197.04	471	0.07	2.22

## Data Availability

All raw and processed sequencing data from this study have been deposited in the CNLR GSA database (https://ngdc.cnLR.ac.cn/gsa (accessed on 28 November 2023)) under the accession number CRA011137.

## Supplementary Materials

The supporting information can be downloaded at https://www.mdpi.com/article/10.3390/ijms25115843/s1.
