# Supplementary material for: Unveiling the Influence of Copy Number Variations on Genetic Diversity and Adaptive Evolution in China’s Native Pig Breeds via Whole-Genome Resequencing
Source: Int J Mol Sci. 2024 May 27;25(11):5843. doi: 10.3390/ijms25115843 (PMC11172908; doi:10.3390/ijms25115843)
Supplement: Supplementary file 1 [file ijms-25-05843-s001.zip › Supplementary Figure 2.pdf]

Supplementary Figure 2

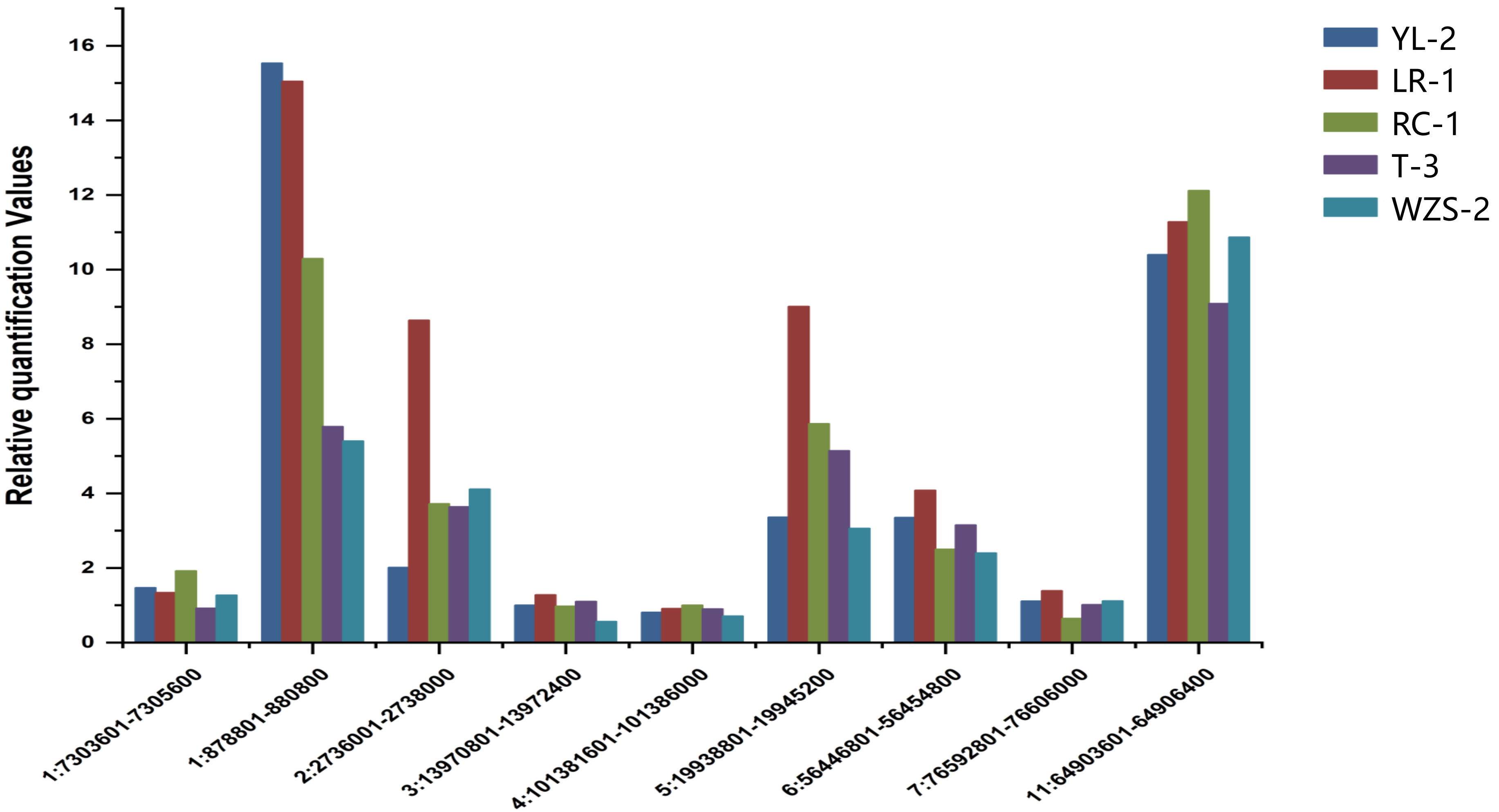

Figure S2. qPCR validation of selected CNVRs. The y-axis shows the Relative quantification Values obtained by qPCR, while the x-axis indicates the sample names in the different CNV regions.
