# Supplementary figures and images for: Unveiling the Influence of Copy Number Variations on Genetic Diversity and Adaptive Evolution in China’s Native Pig Breeds via Whole-Genome Resequencing
Source: Int J Mol Sci. 2024 May 27;25(11):5843. doi: 10.3390/ijms25115843 (PMC11172908; doi:10.3390/ijms25115843)

## Supplementary Figure 1

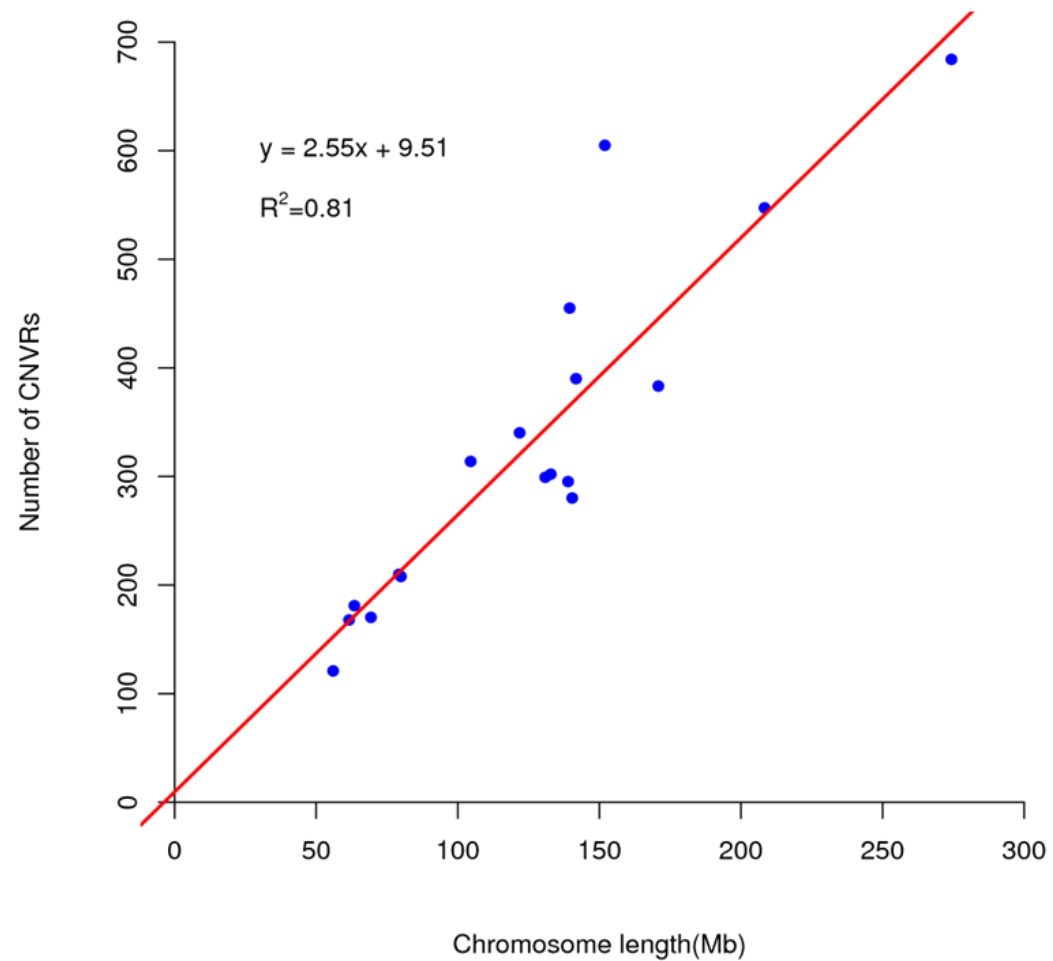

**Figure S1.** Correlation between CNVR counts and chromosome length.

Supplement: Supplementary file 1 [file ijms-25-05843-s001.zip › Supplementary Figure 1.pdf]
